# Anti-CD44 and EGFR Dual-Targeted Solid Lipid Nanoparticles for Delivery of Doxorubicin to Triple-Negative Breast Cancer Cell Line: Preparation, Statistical Optimization, and *In Vitro* Characterization

**DOI:** 10.1155/2022/6253978

**Published:** 2022-07-06

**Authors:** Farnosh Darabi, Massoud Saidijam, Fatemeh Nouri, Reza Mahjub, Meysam Soleimani

**Affiliations:** ^1^Department of Pharmaceutical Biotechnology, School of Pharmacy, Hamadan University of Medical Sciences, Hamadan, Iran; ^2^Department of Pharmaceutics, School of Pharmacy, Hamadan University of Medical Sciences, Hamadan, Iran

## Abstract

**Background:**

Despite being more aggressive than other types of breast cancer, there is no suitable treatment for triple-negative breast cancer (TNBC). Here, we designed doxorubicin-containing solid lipid nanoparticles (SLNs) decorated with anti-EGFR/CD44 dual-RNA aptamers, which are overexpressed in TNBC. For more efficiency in the nuclear delivery of doxorubicin, dexamethasone (Dexa) was chemically attached to the surface of nanoparticles.

**Methods:**

To prepare the cationic SLNs, 6-lauroxyhexyl BOC-ornithine (LHON) was synthesized and was chemically attached to dexamethasone to form Dexa-LHON complexes. The doxorubicin-containing SLNs were prepared via double emulsification (*w*/*o*/*w*) and the solvent evaporation technique. The preparation of SLNs was statistically optimized using the central composite response surface methodology. Independent factors were the GMS/lecithin concentration ratio and the amount of Tween 80, while responses considered were particle size, polydispersity index, and entrapment efficiency of the nanoparticles. The optimized nanoparticles were studied morphologically using transmission electron microscopy, and *in vitro* release of doxorubicin from nanoparticles was studied in phosphate-buffered saline. Then, the designated aptamers were attached to the surface of nanoparticles using electrostatic interactions, and their cytotoxicity was assessed *in vitro*.

**Results:**

The size, PDI, zeta potential, EE%, and LE% of the prepared nanoparticles were 101 ± 12.6 nm, 0.341 ± 0.005, +13.6 ± 1.83 mV, 69.98 ± 7.54%, and 10.2 ± 1.06%, respectively. TEM images revealed spherical nanoparticles with no sign of aggregation. *In vitro* release study exhibited that 96.1 ± 1.97% of doxorubicin was released within 48 h of incubation. The electrostatic attachment of the designated aptamers to the nanoparticles' surface was confirmed by reducing the zeta potential to −15.6 ± 2.07 mV. The *in vitro* experiments revealed that the SLNs/DOX/Dexa/CD44 or EGFR aptamers were substantially more successful than SLNs/DOX/Dexa at inhibiting cell proliferation. Using the MDA-MB-468 cell line, we discovered that SLN/DOX/Dexa/CD44/EGFR aptamers were more effective than other constructs in inhibiting cell proliferation (*p* < 0.001). The reduction of cell viability using this construct suggests that targeting numerous proliferation pathways is effective.

**Conclusion:**

Overall, the finding of this investigation suggested that SLNs/DOX/Dexa/CD44/EGFR could be a promising new enhanced anticancer delivery system and deserved further preclinical consideration.

## 1. Introduction

Triple-negative breast cancer (TNBC) is an aggressive and fatal breast tumor subtype. Although TNBC accounts for only 15%–20% of breast cancer cell types, metastasis and poor prognosis significantly occur in this subtype rather than in the other subtypes [1]. A growing body of evidence suggests that failure in TNBC therapy is mainly due to the inherently aggressive behavior of these cancer cells and the lack of recognized molecular targets for treatment [2]. Another critical challenge of TNBC treatment is intertumor and intratumor heterogeneity on the molecular, clinical, and pathologic levels [3]. Because TNBC is so heterogeneous, finding efficient treatment targets is difficult, and there is currently no approved targeted therapy for the treatment of TNBC. Multiple evidence implies that these characteristics influence chemotherapy resistance, metastasis, and poor clinical outcomes [4]. Therefore, in recent years, combination therapy using chemotherapeutic drugs or using two or more targeted treatments has been assessed by field investigators to improve the treatment efficacy against TNBC [5].

As a unique population of tumor cells, cancer stem cells have been linked to chemoresistance, invasion, and tumor relapse [6]. CD44, a transmembrane glycoprotein, is considered one of the dominant markers on the surface of cancer stem cells in TNBC. Different studies have shown that the CD44 marker is more abundant in TNBC than other breast cancer subtypes. This marker plays an essential role in various cellular pathways, including adhesion, migration, proliferation, motility, and differentiation [7, 8]. The pleiotropic functions of CD44 in cancer could provide a novel biological target for targeted therapy. Different preclinical and clinical trials of the anti-CD44 antibodies have been conducted in malignancies in which overexpression of CD44 is involved [9, 10].

Among several factors involved in tumor formation, angiogenesis is one of the dominant factors in the development of cancers. It is considered an important goal in treating many tumor cells. Epidermal growth factor receptor (EGFR) is a significantly overexpressed protein in TNBC cells [11]. Therefore targeting this pathway with EGFR inhibitors can optimally prevent the growth and metastasis of the TNBC and has been considered in different studies [12, 13].

Over the years, targeted nanocarrier-based drug delivery systems such as liposomes, solid lipid nanoparticles, polymeric micelles, and carbon nanotubes have been used to treat and minimize invasion in several cancer types, sparing healthy cells at the target site. These systems present the potential to enhance the concentration of drugs in the tumor through a mechanism known as enhanced permeability and retention (EPR), which is improved by the application of smaller particles (i.e., 100–200 nm) when compared with those having larger particle sizes [14, 15]. Moreover, by the decoration of specific ligands to the surface of nanocarriers, the cellular uptake of the therapeutics could be facilitated.

Among different types of nanoparticles, solid lipid nanoparticles (SLNs) pose the advantages that are most associated with their ease of manufacturing and commercialization as well as their physical stability, well-protection of the entrapped drug against chemical decomposition, the provision of controlled drug release, and the exceptional acceptability [16]. Compared with the nanoemulsions prepared with liquid lipids, SLNs, due to their solid matrix, have more potential for the controlled release of their cargo [17].

Various approaches have been used to reduce the off-target toxicity of chemotherapy in normal cells. Due to their low molecular weights, lack of immunogenicity, and ease of preparations, RNA aptamers, defined as RNA oligonucleotides that can bind to specific target molecules with high affinity and selectivity, have been applied for tumor target delivery of anticancer agents [18].

To enhance the delivery to the nucleus, the nanoparticles can be surface modified by dexamethasone (Dexa). After cellular uptake of the carrier, dexamethasone, considered an effective synthetic glucocorticoid, could bind to the glucocorticoid receptor and translocate the complexes into the nucleus through the present glucocorticoid receptors in the nuclear envelope (NE) [19].

**Figure 1:**
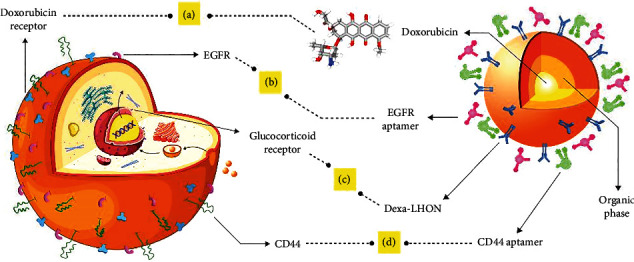
A: doxorubicin encapsulated in solid lipid nanoparticles. B: EGFR is significantly expressed in TNBC and has an essential role in tumor angiogenesis. C: DEXA_LHON, as a ligand affecting the glucocorticoid receptor, facilitates the transport of the nanoparticle into the nucleus. D: CD44 is the most important marker of cancer stem cells.

Given the heterogeneous nature of TNBC, this study was aimed at developing dual-targeted solid lipid nanoparticles encapsulating doxorubicin in TNBC treatment *in vitro*. For cellular targeting of TNBCs, the nanoparticles were electrostatically surface-decorated with anti-CD-44 and EGFR aptamers. Furthermore, for nucleus delivery, the surface of nanoparticles was also covalently modified with dexamethasone molecules (Figure 1). The nanoparticles were optimized using the Box-Behnken response surface methodology, and their antitumor efficacy was studied in the MDA-MB-468 cell line.

## 2. Methods and Materials

### 2.1. Materials

Doxorubicin hydrochloride (DOX) was purchased from Pfizer (New York, United States). Lauric acid, 2-(tert-butoxycarbonyloxyimino)-2-phenylacetonitrile (BOC-ON), L-ornithine, 2-iminothiolane, glycerol monostearate, 3-(4,5-dimethylthiazol-2-yl)-2,5-diphenyltetrazolium bromide (MTT), fetal bovine serum (FBS), penicillin, streptomycin, and trypsin EDTA (0.25%) were all purchased from Sigma-Aldrich (St Louis, USA). Trifluoroacetic acid (CF_3_COOH), 1,4-dioxane, 1,6-hexanediol, LiChrosolv® HPLC grade methanol, LiChrosolv® HPLC grade acetonitrile, Tween 80, dichloromethane, mannitol, trifluoroacetic acid (TFA), sodium hydroxide (NaOH), and sodium dihydrogen phosphate (NaH_2_PO_4_) were all obtained from Merck (Darmstadt, Germany). Dexamethasone was purchased from Kimiadaro (Alborz, Iran). The cDNA synthesis kit and total RNA extraction kit were purchased from BioFACT (South Korea). The T7 transcription kit was purchased from Roboklon (Berlin, Germany). Roswell Park Memorial Institute (RPMI) 1640 medium was purchased from the Beta Gene (Mashhad, Iran). Doubled-distilled water was obtained via the Millipore Milli-Q® water purification system (Massachusetts, United States). All other chemicals and reagents were of pharmaceutical grade and used as received.

### 2.2. Pharmaceutical Tests

#### 2.2.1. Chemical Synthesis of Dexa-LHON Complexes

Dexa-LHON complexes were synthesized according to the previously published method with minor modification [20]. Briefly, one equivalent of L-ornithine was stirred with 2.5 equivalents of BOC-ON in wet 1,4-dioxane at room temperature overnight to produce BOC-ornithine. Then, one equivalent of the synthesized BOC-ornithine was esterified with 5 equivalents of 1,6-hexanediol to form 6-hydroxyphenyl BOC-ornithinate. The compound was condensed with one equivalent of lauric acid to generate 6-lauroxyhexyl BOC-ornithine (LHON). The resulting LHON was bound to dexamethasone via Traut's reagent to form the Dexa-LHON complex. A 45 *μ*m filter removed the insoluble impurities, and the resulting compound was dialyzed against distilled water for further purification. The chemical attachment of dexamethasone to LHON was confirmed via FT-IR spectroscopy.

For determination of the efficacy of the synthesis, the amount of unbound dexamethasone was analyzed using an HPLC method. A Shimadzu® liquid chromatography system, equipped with an LC-20AD pump and a UV-visible detector that was set at 254 nm, was used for chromatography. The mobile phase consisted of a mixture of acetonitrile (51% *v*/*v*) and deionized water (49% *v*/*v*) containing TFA (0.1% *v*/*v*), and the stationary phase was a Hector-M® ODS column (250∗4.6 mm). The flow rate was kept constant at 1 ml/min. The retention time for dexamethasone was 6.18 min, and the method was proven to be linear in the range of 1 *μ*g/ml to 100 *μ*g/ml with a regression coefficient of *R*^2^ = 0.99. The intra- and interday precision and accuracy were calculated and well-agree with the specified ranges in the appropriate ICH guidelines. The complexation efficiency of dexamethasone was calculated according to the following equation:

(1) $$\begin{array}{c} \text{Complexation efficiency}=\frac{\text{total amount of Dexa}-\text{amount of unbound Dexa}}{\text{total amount of Dexa}}*100. \end{array}$$

#### 2.2.2. Preparation of Solid Lipid Nanoparticles (SLNs)

SLNs were prepared following the double emulsification (*W*_1_/*O*/*W*_2_) and solvent evaporation technique [21]. To prepare the first aqueous phase (i.e., *W*_1_), doxorubicin (0.5 ml) was dissolved in 1.5 ml of double-distilled water. Separately, the organic phase was prepared by complete dissolution of glycerol monostearate (GMS) (10 mg) and various amounts of soy lecithin in 5 ml of dichloromethane. To prepare for the second aqueous phase (*W*_2_), Dexa-LHON (7.6 mg), which was previously synthesized, was added to a different amount of Tween 80. The mixture was dissolved entirely in doubled-distilled water. All solutions, including the first and second aqueous and organic phases, were heated to 55°C using a Memmert® bain-marie (Germany).

For the preparation of the first emulsification step (i.e., *W*_1_/*O*), the first aqueous phase was added dropwise to the organic phase under homogenization (10,000 rpm) using a Heidolph® homogenizer (Schwabach, Germany). The mixture was kept homogenized for an additional 10 min, followed by the second emulsification step (i.e., *W*_1_/*O*/*W*_2_). The resultant emulsion was added dropwise to the second aqueous phase under homogenization (12,000 rpm). The mixture was kept stirred for an additional 15 min. The organic solvent (i.e., dichloromethane) was evaporated entirely using a rotary evaporator. Finally, the colloidal suspension was centrifuged at 20,000 rpm for 30 min (at 4°C), and the settled down nanoparticles were obtained and collected. At the same time, the transparent supernatant was analyzed for calculation of entrapment efficiency (EE%) and loading efficiency (LE%).

#### 2.2.3. Experimental Design

A three-leveled, two-factor central composite response surface methodology was applied for statistic optimization of the nanoparticles. Independent variables (i.e., factors) were considered as GMS/lecithin concentration ratio (A) and the amount of Tween 80 (B), while the dependent variables (i.e., responses) were particle size (*Y*_1_), PDI (*Y*_2_), and entrapment efficiency (EE%) (*Y*_3_). The ranges and constraints are summarized in Table 1. The generation and appraisal of the experimental design were done using Design-Expert® software (Version 7, Stat-Ease Inc., Minneapolis, MN, USA). The software suggested 11 experiments and was experimentally prepared in triplicate. The experimental design suggested by the software and their related experimental responses is shown in Table 2.

#### 2.2.4. Determination of the Particle Size and Zeta Potential

The mean particle diameter was measured by dynamic light scattering (DLS) using photon correlation spectroscopy via a Zetasizer Nano ZS (Malvern Instruments, Worcestershire, UK) at a fixed angle of 90° and 25°C. Samples of the prepared SLNs were diluted in 1 : 9 in double-distilled water up to counting 50–300 kcps. The reported data were particle size and polydispersity index (PDI). Moreover, zeta potential measurements were carried out using the same instrument at a temperature of 25°C, and the data were reported as millivolt (mV). All measurements were done in triplicate.

#### 2.2.5. Determination of the Entrapment Efficiency (EE%) and Loading Efficiency (LE%)

The indirect method was applied to determine EE% and LE% in this study. Briefly, freshly prepared colloidal nanosuspensions were centrifuged, and the transparent supernatant was analyzed for unentrapped doxorubicin using a Shimadzu® HPLC system equipped with an LC-20AD pump and an RF20A fluorescence detector. The excitation and emission wavelengths were set as 475 nm and 555 nm, respectively. A Hector-M® ODS column (250∗4.6 mm) was used as the stationary phase, and the analysis was performed at ambient temperature. The mobile phase consisted of a mixture of acetonitrile (45% *v*/*v*) and deionized water (55% *v*/*v*), and the flow rate was set as 1 ml/min. The peak of DOX appeared at 2.8 min, and the method was proven to be linear in the range of 1 *μ*g/ml to100 *μ*g/ml with a regression coefficient of *R*^2^ = 0.9960. The intra- and interday precision and accuracy were calculated separately, and the calculated values well-agreed with the specified ranges in the appropriate ICH guidelines. The EE% and LE% of doxorubicin in the nanoparticles were calculated according to Equations (2) and (3), respectively as follows:

(2) $$\begin{array}{c} \text{EE}\%=\frac{\text{Total amount of DOX}-\text{unentrapped DOX }}{\text{Total amount of DOX}}*100, \end{array}$$

(3) $$\begin{array}{c} \text{LE}\%=\frac{\text{Total amount of DOX}-\text{unentrapped DOX}}{\text{Total weight of nanoparticles}}*100. \end{array}$$

#### 2.2.6. Lyophilization

The prepared SLNs were lyophilized using a programmable freeze dryer (Shin PVTFD10R, Shinil Lab, Korea). To preserve the size of particles and improve the chemical stability, mannitol as a cryoprotectant was added to the SLN dispersion before freezing. Slow freezing was carried out on the shelves in the freeze dryer (shelf temperature −40°C). The samples were lyophilized for 24 h from −40°C to 25°C at an increasing rate of 5°C/h. Lyophilized products were reconstituted via sonication (2 min, 500 W, POWERSONIC 510, Korea).

#### 2.2.7. *In Vitro* Release of Doxorubicin from SLNs

The release profile of DOX from the optimized SLN formulations was determined using the dialysis bag method [22] via a cellulose membrane dialysis tube with a molecular weight cut off 10,000 Da (SLS Co., UK). The appropriate amount of the lyophilized nanoparticles, equivalent to 2 mg of doxorubicin, was dispersed in doubled-distilled water and instilled in a dialysis bag. The dialysis bag was sealed at both ends and immersed in 250 ml phosphate-buffered saline (pH = 7.4). Considering the aqueous solubility of doxorubicin (i.e., 0.5 mg/ml), the establishment of the sink condition was ascertained.

The experiment was conducted in a thermo-stated shaking water bath (Memmert, ONE10, Germany). The temperature was kept constant at 37 ± 0.5°C, and the agitation rate was 200 rpm. Withdrawing of samples (1 ml) was performed at predetermined time intervals following replenishment with an equal volume of the preheated, fresh release medium. The amount of DOX in the collected samples was analyzed using the previously developed HPLC method. All measurements were performed in triplicate.

#### 2.2.8. Transmission Electronic Microscopy (TEM)

The morphology of the optimized and SLN formulation was examined by transmission electron microscopy (TEM) (EM10C; Zeiss, Jena). The SLN dispersion was fixed in a coated copper grid (300 mesh using osmium tetroxide, dried for 24 h, and finally analyzed at an acceleration voltage of 80 kV).

### 2.3. Biotechnology Tests

#### 2.3.1. Construction of RNA Aptamers

CD44 and EGFR aptamers were individually produced using *in vitro* transcription with the PCR product as a template.

The CD44 aptamer (5′GGGATGGATCCAAGCTTACTGGCATCTGGATTTGCGCGTGCCAGAATAAAGAGTATAACGTGTGAATGGGAAGCTTCGATAGGAATTCGGAAAATTCTTTGGTCTGCATTCACATCA-3′) was synthesized by the Bio Basic Company (Canada) as a PCR template. PCR was performed with the forward primer (5′-GAAATTAATACGACTCACTATAGGGATGGATCCAAGC-3′) and reverse primer (5′-TGATGTGAATGCAGACCAAAGAATTTTCCGAATTCC-3′).

The EGFR aptamer (5′-GCCTTAGTAACGTGCTTTGATGTCGATTCGACAGGAGGCAAAAATGTGAATGCAGACCAAAGAATT-3′) was synthesized by the Bio Basic Company (Canada) as a PCR template. PCR was performed with the forward primer (5′-GAAATTAATACGACTCACTATAGGGCCTTAGTAACG-3) and reverse primer (5′-AATTCTTTGGTCTGCATTCACATTTTTG-3′).

The forward primers in two templates contain the T7 RNA polymerase promoter binding sequence, which is underlined.

Transcription was performed with highly pure PCR products as templates using the APT-GET T7 transcription kit (Roboklon, Germany), following the manufacturer's instructions. After *in vitro* transcription, DNA templates were degraded by adding proper amounts of DNase1 enzyme (37°C for 15 minutes), and RNA aptamers were purified using ammonium acetate/ethanol precipitation. Briefly, a volume of 5 M ammonium acetate was added to the mixture, mixed well, and placed on ice for 15 minutes. The mixture was centrifuged at 14,000 rpm for 20 minutes at 4°C. After centrifugation, the supernatant was gently discarded, and the pellet was washed with 70% cold ethanol. The mixture containing pellets and ethanol was centrifuged for 10 minutes at 10°C at 14,000 rpm. After discarding the supernatant, the microtubes were placed at room temperature to evaporate the remaining ethanol for a few minutes. The remaining pellet was dissolved in free nuclease water, and the resulting solution was kept at -20°C. The DNA and RNA concentrations were determined via absorbance at 260 nm using a NanoDrop 2000 UV-Vis spectrophotometer (Thermo Scientific, USA).

#### 2.3.2. Binding of Aptamers to the Surface of Nanoparticles

The positive surface charges of the prepared SLNs were electrostatically attached to anti-EGFR and CD44 RNA aptamers. The attachment was confirmed by measuring the size and zeta potential of the nanoparticles. 0.25 *μ*M of each aptamer was used to decorate the surface of nanoparticles.

### 2.4. MTT Assay

The MDA-MB-468 cells (Pasture, Iran) were cultured at 37°C in 5% CO_2_ and 95% humidified air in RPMI containing 10% FBS, 100 units/ml penicillin, and 100 *μ*g/ml streptomycin. In summary, 8 × 10^3^ cells/well were seeded in flat-bottom 96-well plates and allowed to attach overnight. Then, cells were treated with various concentrations of prepared compounds. 48 h afterward, 20 *μ*l of MTT solution (5 mg/ml) was added to each well. The plates were then incubated for 4 hours. After removing the medium, 100 *μ*l of DMSO solution was added to each well to dissolve the resulting formazan. After 10 minutes, the optical absorption (OD) was read via a plate reader at 570 nm.

### 2.5. Statistical Analysis

All studies were repeated three times, and all measurements were carried out in triplicate. Results were reported as means ± SEM. Statistical significance was analyzed using Student's *t*-test and one-way analysis of variance (ANOVA). Differences between experimental groups were considered significant when the *p* value was less than 0.05 (*p* < 0.05).

## 3. Results

### 3.1. Characterization of Dexa-LHON Complexes

**Figure 2:**
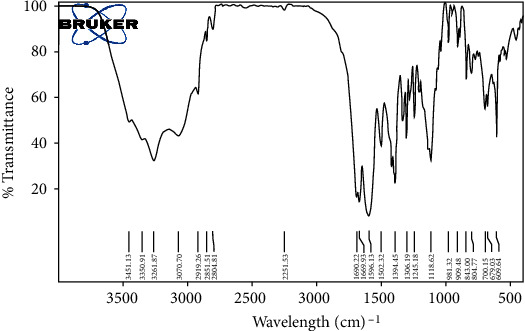
FT-IR spectrum of Dexa-LHON. The complexation efficiency of dexamethasone in the prepared complex was calculated by the indirect method and reported as 86.5 ± 6.47%.

In this study, 6-lauroxyhexyl BOC-ornithine (LHON) was synthesized and was chemically complexed with dexamethasone by covalent bonds. The FT-IR spectrum of Dexa-LHON is illustrated in Figure 2. The complexation efficiency of dexamethasone in the prepared complex was calculated via the indirect method and reported as 86.5 ± 6.47%.

### 3.2. Preparation of Solid Lipid Nanoparticles

As shown in Table 2, the preparation of 11 experimental formulations was adequate for optimization via the central composite response surface methodology. The experimentally obtained data were statistically analyzed using ANOVA. The regression method fits the data to appropriate mathematical models explained by polynomial equations. Each fitted model was characterized by statistical parameters (i.e., *R*^2^, adjusted-*R*^2^, predicted-*R*^2^, and adequate precision). The effect of each independent variable (i.e., factors) and their binary interactions on the dependent variables (i.e., responses) was represented with 3D response surface plots. Finally, the software predicted the optimized formulation parameter to minimize both particle size and polydispersity index values and maximize EE%.

#### 3.2.1. Particle Size

**Figure 3:**
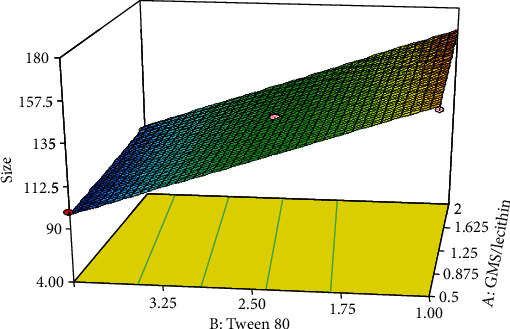
Response 3D plots for alteration in size of nanoparticles. The size of nanoparticles increased slightly by increasing the concentration ratio of GMS/lecithin from 0.5% (*w*/*v*) to 2% (*w*/*v*). On the other hand, by increasing the concentration of Tween 80 from 1% (*w*/*v*) to 4% (*w*/*v*), nanoparticles' size declined linearly.

As summarized in Table 2, the particle size of SLN formulations varied from 69.71 nm to 214.7 nm. ANOVA statistically analyzed the results to identify the best significant model for predicting average particle diameter. The characteristics of the best-fitted linear proposed model are summarized in Table 3. As presented in the table, the proposed significant model (*p* < 0.001) exhibited proper characteristics and a nonsignificant lack of fit (*p* > 0.5), which indicates the appropriate predictability of the model. Analysis of variance for the proposed model revealed that linear coefficients of GMS/lecithin (*X*_1_) and concentration of Tween 80 (*X*_2_) are significant (*p* < 0.001). The coefficients of the significant variable on the particle size are shown in the following equation:

(4) $$\begin{array}{c} Y_{1}=+159.28881+3.58891*X_{1}-20.93034*X_{2}, \end{array}$$

where *Y*_1_ is the average particle diameter, *X*_1_ is the linear coefficient for the concentration ratio of GMS/lecithin, and *X*_2_ is the linear coefficient for the concentration of Tween 80. The 3D response surface plot for changes in particle size of the prepared solid lipid nanoparticles as the result of alteration in the concentration of Tween 80 is shown in Figure 3. As shown in the figure, the size of nanoparticles increased slightly by increasing the concentration ratio of GMS/lecithin from 0.5% (*w*/*v*) to 2% (*w*/*v*). On the other hand, by increasing the concentration of Tween 80 from 1% (*w*/*v*) to 4% (*w*/*v*), nanoparticles' size declined linearly.

#### 3.2.2. Polydispersity Index (PDI)

PDI is considered an index that shows the homogeneity of nanoparticles. In this study, the PDI value in different formulations ranged from 0.191 to 0.637 (Table 2). To identify the best significant model for predicting the polydispersity index of the nanoparticle diameter, the results were statistically analyzed via ANOVA. The characteristics of the best-fitted quadratic proposed model are summarized in Table 3. As shown in the table, the proposed significant model (*p* < 0.01) exhibited proper characteristics and a nonsignificant lack of fit (*p* > 0.5), which indicates adequate predictability for the model.

**Figure 4:**
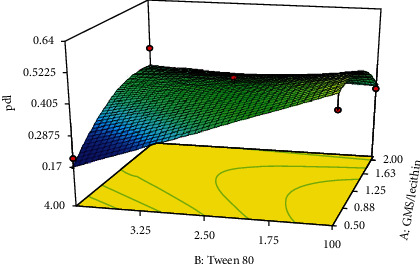
Response 3D plots for alteration in polydispersity index of the nanoparticles. In the lowest concentration ratio of GMS/lecithin (i.e., 0.5), by increasing the concentration of Tween 80, the PDI values of the nanoparticles sharply declined. However, in the highest concentration of GMS/lecithin (i.e., 2.0), a slight decrease in PDI values was observed due to a rise in the concentration of Tween 80.

Analysis of variance for the proposed model revealed that the linear coefficient of the concentration of Tween 80 (*X*_2_) as well as the squared coefficient of the concentration ratio of GMS/lecithin (*X*_1_^2^) was significant (*p* < 0.03). Moreover, it was found that the binary interaction between the two factors *X*_1_ and *X*_2_ was also significant (*p* < 0.04). The coefficients of the significant variable on the polydispersity index of the nanoparticles are shown in the following equation:

(5) $$\begin{array}{c} Y_{2}=+0.59450+0.10902*X_{1}-0.15074*X_{2}-0.12740{X_{1}}^{2}+0.082000*X_{1}\cdot X_{2}, \end{array}$$

where *Y*_2_ is the polydispersity index of the solid lipid nanoparticles, *X*_1_ is the linear coefficient for the concentration ratio of GMS/lecithin, *X*_2_ is the linear coefficient of the concentration of Tween 80, and *X*_1_ · *X*_2_ is the binary interaction coefficient between *X*_1_ and *X*_2_. The 3D response surface plot for changes in polydispersity index due to alteration in the concentration ratio of GMS/lecithin and concentration of Tween 80 is shown in Figure 4. As shown in the figure, in the lowest concentration ratio of GMS/lecithin (i.e., 0.5), by increasing the concentration of Tween 80, the PDI values of the nanoparticles sharply declined. However, in the highest concentration of GMS/lecithin (i.e., 2.0), a slight decrease in PDI values was observed due to rising the concentration of Tween 80.

Moreover, as depicted in the figure, in the lowest concentration of Tween 80 (i.e., 1%), the PDI of the prepared nanoparticles was increased by increasing the concentration ratio of GMS/lecithin. On the other hand, in the highest concentration of Tween 80 (i.e., 4.0), although the PDI values of the nanoparticles were increased by increasing the concentration ratio of GMS/lecithin from 0.5 to 1.25, but by further increasing the concentration ratio up to 2.0, the PDI values were observed to decrease.

#### 3.2.3. Entrapment Efficiency (EE%)

**Figure 5:**
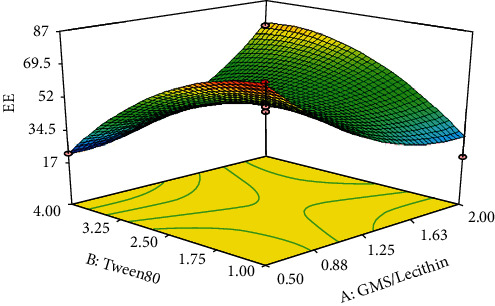
Response 3D plots for alteration in the EE% values. In the lowest concentration of Tween 80 (i.e., 1%), the values for EE% fell dramatically by increasing the concentration ratio of GMS/lecithin from 0.5 to 1.25%. Further increase in the concentration ratio up to 2.0 resulted in a slight and nonsignificant reduction in the EE%. On the other hand, in the highest concentration of Tween 80 (i.e., 4.0), the values for EE% of the nanoparticles were increased by increasing the concentration ratio.

The EE% of the DOX in various formulations of the nanoparticles varied from 17.67% to 86.72% (Table 2). For better statistical model fitting and regression, the obtained data for EE were transformed to a base-10-logarithm. To identify the best significant model for predicting the logarithm of EE%, the results were statistically analyzed via ANOVA. The characteristics of the best-fitted quadratic proposed model are summarized in Table 3. As presented in the table, the proposed significant model (*p* < 0.03) exhibited proper characteristics and a nonsignificant lack of fit (*p* > 0.2), which indicates adequate predictability of the model. Analysis of variance for the proposed model revealed that the squared coefficient of the concentration of Tween 80 (*X*_2_) as well as its binary interaction with the concentration ratio of GMS/lecithin (*X*_1_) was significant (*p* < 0.02). The coefficients of the significant variables on the entrapment efficiency of the nanoparticles are presented in the following equation:

(6) $$\begin{array}{c} \text{Log}Y_{3}=+2.35050-0.96358*X_{1}+0.014851*X_{2}–0.070603*{X_{2}}^{2}+0.23627*X_{1}\cdot X_{2}+0.14905{X_{1}}^{2}, \end{array}$$

where *Y*_3_ is the entrapment efficiency of the nanoparticles, *X*_1_ is the linear coefficient for the concentration ratio of GMS/lecithin, *X*_2_ is the linear coefficient of the concentration of Tween 80, *X*_2_^2^ is the squared coefficient of the concentration of Tween 80, and *X*_1_ · *X*_2_ is the binary interaction coefficient between *X*_1_ and *X*_2_. The 3D response surface plot of the variation in EE% due to changes in independent variables is exhibited in Figure 5. As shown in the figure, in the lowest concentration of Tween 80 (i.e., 1%), the values for EE% fell dramatically by increasing the concentration ratio of GMS/lecithin from 0.5 to 1.25%. Further increase in the concentration ratio up to 2.0 resulted in a slight and nonsignificant reduction in the EE%. On the other hand, in the highest concentration of Tween 80 (i.e., 4.0), the values for EE% of the nanoparticles were increased by increasing the concentration ratio.

Moreover, as depicted in Figure 5, although in the lowest value for a concentration ratio of GMS/lecithin (i.e., 0.5), the EE% was decreased as the result of the increasing concentration of Tween 80, in the highest concentration ratio of GMS/lecithin (i.e., 2.0), a sharp increase in the values of EE% could be observed following increase of the concentration of Tween 80.

#### 3.2.4. Optimization and Model Validation

The optimization of the characteristics of the prepared solid lipid nanoparticles was performed using statistical and mathematical analyses of the obtained data. The suggested optimized factors and their corresponding predicted responses are depicted in Table 4. As shown in the table, the suggested optimized values for the concentration ratio of GMS/lecithin (*X*_1_) and concentration of Tween 80 were 2.0 and 3.73% (*w*/*v*), respectively. It is predicted that the prepared nanoparticles exhibit the minimized particle size, the minimized PDI, and the maximized EE% in the optimized conditions. To determine the predictability and reliability of the proposed models, the nanoparticles were experimentally prepared and characterized (*n* = 3). For each response, the prediction error (%) was calculated according to the following equation:

(7) $$\begin{array}{c} \text{Prediction Error }\left(\%\right)=\frac{\text{Observed Response}-\text{Suggsted Response}}{\text{Observed Response}}*100. \end{array}$$

The experimentally observed responses and the appropriate calculated prediction error (%) are summarized in Table 5. The table shows that all calculated prediction errors (%) were well below 15%, indicating the fitted models' high predictability and reliable characteristics.

### 3.3. Morphological Studies

**Figure 6:**
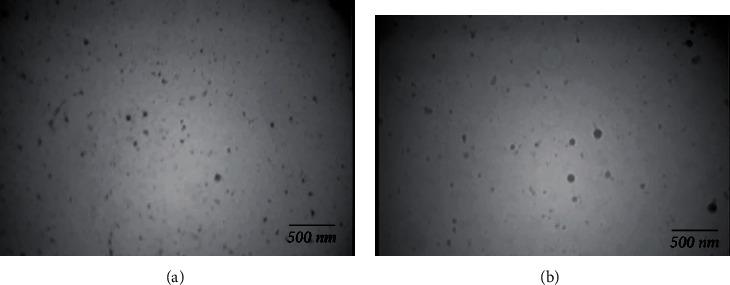
TEM images: (a) SLN/Dox; (b) SLn/Dox/Dexa. The images indicate the formation of spherical-shaped nanoparticles.

The lyophilized, optimized nanoparticles were studied morphologically using transmission electron microscopy (TEM). The particles were fixed with a solution of osmium tetroxide at 0.4% for 20 min and dried at room temperature for 24 h before imaging. The obtained microscopic images are exhibited in Figure 6. The images indicate the formation of spherical-shaped nanoparticles. No sign of aggregation was observed in the colloidal suspension. The figures show that the particle size, determined from the microscopic images, was well in accordance with the values determined using photon correlation spectroscopy (PCS).

### 3.4. In Vitro Release

**Figure 7:**
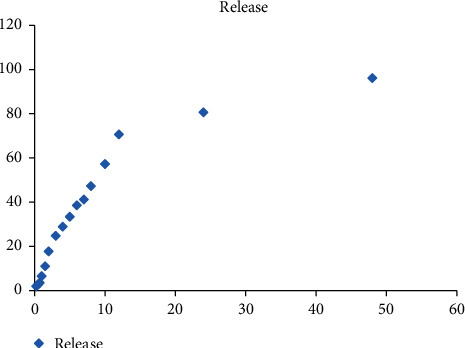
The in vitro release profile (*n* = 3). In the first hour of incubation, the formulation exhibited a slow release rate, and only 6.5 ± 0.25% of the doxorubicin was released after 60 min. However, the release rate increased by increasing the incubation time, and finally, 96.16 ± 1.97% of the drug was released after 48 hours.

The profile of *in vitro* release of doxorubicin from the optimized SLN formulation is shown in Figure 7. As shown in the figure, in the first hour of incubation, the formulation exhibited a slow release rate, and only 6.5 ± 0.25% of the doxorubicin was released after 60 min. However, the release rate increased by increasing the incubation time, and finally, 96.16 ± 1.97% of the drug was released after 48 hours.

### 3.5. Construction and Characterization of CD44 and EGFR Aptamers

CD44 and EGFR RNA aptamers are produced via *in vitro* transcription using PCR products as a DNA template (Table 6). 2′-Fluoropyrimidines were integrated into two aptamer chains during transcription to improve serum stability. Briefly, following confirmation of the synthesis of two sequences in PCR, purification of the resulting products was performed using a PCR cleanup kit and then using phenol-chloroform extraction and ethanol precipitation to obtain a suitable amount of very pure DNA with a high concentration suitable for achieving *in vitro* transcription steps. An appropriate amount of DNase1 enzyme was added to the reaction mixture and placed at 37°C for 15 minutes to destroy the DNA template. After in vitro transcription, RNA strands were purified using ammonium acetate precipitation, and it was confirmed through gel electrophoresis.

### 3.6. Binding of Aptamers to the Surface of Nanoparticles

The negatively charged aptamer can establish electrostatic interactions with the surface of the positively charged solid lipid nanoparticles. The electrostatic attachment of the aptamer to the surface of nanoparticles was confirmed via a significant reduction in the zeta potential of nanoparticles from +13.6 ± 1.83 mV to −15.6 ± 2.07 mV (Table 7). Moreover, as shown in the table, the electrostatic conjugation of the aptamer to the surface of nanoparticles caused a significant increase in particle size.

### 3.7. MTT Assays

**Figure 8:**
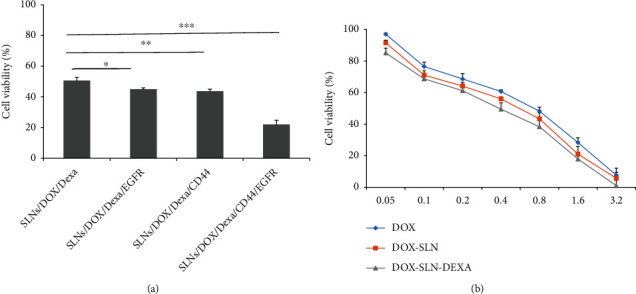
Evaluation of the cytotoxicity of the different formulations after 48 h incubation with MDA-MB-468 cell line. (a) Cytotoxicity comparison between DOX, SLNs/DOX, and DOX-SLNs-Dexa. (b) Cytotoxicity comparison between SLNs/DOX/Dexa with SLNs/DOX/Dexa/EGFR, SLN/Dox/Dexa/CD44, and SLNs/DOX/Dexa/CD44/EGFR. SLNs/DOX/Dexa/CD44/EGFR are substantially more effective in reducing the cell line viability (*n* = 3, ^∗^*p* < 0.05, ^∗∗^*p* < 0.01, and ^∗∗∗^*p* < 0.001).

The MTT assay detected the antiproliferative impact of different Dox formulations on the MDA-MB-468. The MDA-MB-468 is positive for EGFR and CD44 [23, 24]. The breast cancer cell line was treated with an increasing concentration of varying Dox formulations. They all had dose-dependent cellular toxicity on the MDA-MB-468 (Figure 8(a)). The IC_50_ value of SLNs/DOX/Dexa was calculated (0.34 *μ*M) and used for the following cytotoxicity treatment. In comparison with SLNs/DOX, Dexa and SLNs/DOX/Dexa/CD44 reduced the viability of the cancer cell line significantly (*p* < 0.01). In addition, in comparison with SLNs/Dox/Dexa, exertion of more cytotoxicity was observed using EGFR-decorated SLNs/DOX/Dexa (*p* < 0.05). After that, the simultaneous decoration of SLNs/DOX/Dexa via EGFR and CD44 aptamers was considered. As shown in Figure 8(b), SLNs/DOX/Dexa/CD44/EGFR are substantially more effective in reducing the cell line viability (*p* < 0.001).

## 4. Discussion

Breast cancer is one of the most common causes of death in women worldwide. TNBC is the most aggressive kind among these malignancies [1, 25]. In this study, to enhance the delivery of doxorubicin as an antitumor agent to TNBC cells, a solid lipid nanoparticle (SLN) was synthesized. To promote binding avidity and internalization of SLNs, specific aptamers (i.e., anti-CD-44 and EGFR aptamers) were attached to the surface of nanoparticles. Moreover, the nanoparticles were surface-decorated by a conjugated cationic lipid (Dexa-LHON) to facilitate subcellular nuclear delivery. Dox is a potent anticancer agent and a topoisomerase II inhibitor, but its long-term clinical application is associated with cardiomyopathy and other severe systemic toxicities [26]. Recent studies proved that DOX molecules encapsulated in long-circulating liposomes exert strong inhibitory effects on tumor growth and reduce adverse events [27].

The results of various studies show that the application of SLNs for the treatment of TNBC is more effective than the nonencapsulated free drug. Eskiler et al. exhibited that talazoparib-containing SLNs could significantly suppress MDR1, BCRP, and MRP1 genes and their protein expression levels rather than free talazoparib [28]. According to the findings, talazoparib-SLNs can be considered a potential therapeutic carrier for reversing multidrug resistance in TNBC. In line with the hypothesis that the entrapment of the therapeutic molecules within SLNs improved the cellular cytotoxicity of DOX, the results of this study indicated the enhanced cytotoxic effects of DOX-SLNs compared with free SLNs. Various cationic lipids such as CTAB (cetyltrimethylammonium bromide) [29] and DDAB (dimethyldioctadecylammonium bromide) have been introduced for the preparation of lipid-based nanoparticles. However, toxicity is still an obstacle to the use of cationic lipids. Some researchers have indicated that cationic lipids bearing ester bonds are more biodegradable and, therefore, are associated with less cytotoxicity [30]. Tang and Hughes demonstrated the lower toxicity of 6-lauroxyhexyl BOC-ornithine (LHON) as a cationic lipid [20].

This study showed that the size of nanoparticles would decrease by elevation of the concentration of Tween 80 (i.e., surfactant). It is postulated that by increasing the concentration of the surfactant, the surface tension at the interface of the aqueous and oil phase would be reduced. Therefore, the homogenization of the two phases (i.e., oil and aqueous phase) could be facilitated, and consequently, the size of nanoparticles would decline. In the low levels of the surfactant concentration, the interface of the two phases is not entirely covered. Therefore, the droplets would coalesce, increasing the diameter of nanoparticles. However, by increasing the concentration of Tween 80, many surfactant molecules can be absorbed on the interface of oil and water surfaces, reducing surface tension. Moreover, the elevation of the concentration of surfactant can cause an increase of the absorption rate of the surfactant to emulsified droplets, leading to a slight decrease in the size of nanoparticles [31]. In agreement with the obtained results in the studies performed by Bąk and Podgórska [32], it was shown that by elevation of the concentrations of either Tween 20 or Tween 80, the particle size of the prepared solid lipid nanoparticles was decreased.

The results revealed that the PDI values declined by increasing the surfactant concentration. Similar to this study, Kaur et al. [33] reported a decrease in PDI values due to elevation of the surfactant level. Moreover, it was observed that the PDI was increased by increasing the concentration ratio of GMS/lecithin at high surfactant levels. Similarly, in low surfactant levels, increasing the concentration ratio of GMS/lecithin from 0.5 to 1.25 caused an increase in the PDI of nanoparticles. However, it was observed to decrease by further elevation of the concentration ratio. In general, it is reported that by increasing the amount of lipid, the PDI values of the nanoparticles would be increased [34]. Yeganeh et al. [35] reported that by increasing the concentration ratio of GMS/lecithin, the PDI values were increased, but a slight decline in PDI values was also observed in high amounts of lipid.

This study showed that although at high surfactant levels, the entrapment efficiency (EE%) was significantly improved by increasing the concentration ratio of GMS/lecithin, at low surfactant levels, the EE% declined by increasing the ratio. It is suggested that although by increasing the lipid content, more space would be available for entrapping the drug molecules, the hydrophilic characteristics of doxorubicin may prevent the entrapment of the drug molecules inside the solid lipid nanoparticles. It is postulated that the presence of surfactant molecules in high concentrations may increase the drug solubilization into the lipid core of the particles. Therefore, the entrapment efficiency would be increased by the elevation of the lipid content. However, in the low levels of Tween 80, the solubilization ability of the surfactant would be reduced, and therefore, by increasing the concentration ratio of GMS/lecithin, the entrapment of the hydrophilic doxorubicin in the lipid core of the nanoparticles would be reduced. Moreover, it was observed that in high levels of GMS/lecithin, the entrapment of doxorubicin into the nanoparticles would increase by increasing the concentration of the surfactant. The phenomenon can be justified by considering the dominant solubilization ability of the surfactant in higher concentrations which caused the enhanced entrapment of doxorubicin, as a hydrophilic compound, into the lipid core of the prepared solid lipid nanoparticles. Similarly, Hao et al. [36] found that the entrapment efficiency of the hydrophilic compounds can increase via the elevation of the concentrations of both the lipid and surfactant. On the other hand, in low levels of GMS/lecithin, the reduction in EE% due to increase of the surfactant concentration can be attributed to the reduction in the size of nanoparticles. As discussed previously, in low lipid concentrations, the size of nanoparticles was significantly decreased by increasing the surfactant concentration in low levels of GMS/lecithin. Therefore, less space would be available for the entrapment of doxorubicin molecules. This phenomenon leads to a reduction in the entrapment efficiency of the nanoparticles [37].

Morphological studies revealed the preparation of spherical nanoparticles with mean diameters of approximately 99 nm and narrow size distribution. It was reported previously that due to the enhanced permeability and retention (EPR) effect, the spherical particles with a diameter below 200 nm were preferentially accumulated in tumor tissues.

The in vitro release of DOX from the prepared solid lipid nanoparticles follows a biphasic profile, characterized by a rapid initial burst release until 12 h of the incubation period, followed by a slower release till the end of the study (i.e., 50 h). It is suggested that the release of drug molecules that were adsorbed to the surface of nanoparticles was responsible for the observed initial burst release. In contrast, the drug molecules encapsulated inside the particles posed a slower release rate. Previously, the two-phase release behavior has been reported for solid lipid nanoparticles [38].

In this study, it is proposed that after cellular uptake of the nanoparticles, dexamethasone (Dexa) as a synthetic glucocorticoid, which was covalently bound to the solid lipid nanoparticles, could bind to the glucocorticoid receptors (GR), which are expressed in the nuclear envelope and facilitate the translocation of the payload into the nucleus.

In recent years, many studies have used the RNA aptamer to prepare nanoparticle-based targeted drug delivery formulations. Therefore, there is a demand for an efficient and cost-effective approach to RNA aptamer production. Serum stability of the RNA aptamer is another problem for applying aptamers in cancer therapy. Herein, in vitro transcription driven by the T7 RNA polymerase was used for RNA aptamer production and incorporating 2′-fluoropyrimidines in RNA sequences. The cytotoxicity assay results confirmed the production of a stable RNA aptamer using *in vitro* transcription.

CD44 represents a common biomarker of cancer stem cells and promotes epithelial-mesenchymal transition. Much evidence shows that CD44 is a crucial molecule involved in inferior prognosis and tumor metastasis in TNBC cancers. CD44 regulates diverse vital signaling pathways that modulate cancer proliferation, invasion, metastasis, and therapy. Due to pleiotropic roles in carcinoma, CD44 can be considered a new molecular target for therapeutic intervention [39]. Alshaer et al. [40] demonstrated that the conjugation of an anti-CD44 aptamer to liposomes had shown a promising specific drug delivery system. Furthermore, Nabil et al. [41] reported that CD44-targeted polymeric nanoparticles containing momelotinib showed enhanced drug delivery and higher accumulation in TNBC rather than the free drug. Consistent with this result, our investigation indicated that SLN/DOX/Dexa/CD44 formulation had more cytotoxicity than SLNs/DOX/Dexa.

On the other hand, overexpression of EGFR has been reported in up to 78% of TNBC patients, suggesting a therapeutic target for TNBC [42]. It has been shown that the EGFR aptamer could effectively target the uptake of nanoparticles to EGFR-expressing cells. Agnello et al. [43] investigated the efficiency of anti-EGFR and aptamer-decorated nanostructure in TNBC. They showed that, compared with free cisplatin, polymeric nanoparticles conjugated with anti-EGFR aptamer exhibited greater efficiency in tumor targeting and increased therapeutic outcomes. In line with this finding, our research found that the SLN/DOX/Dexa/EGFR formulation was more cytotoxic than the SLN/DOX/Dexa/EGFR formulation.

Most nanoparticulate formulations for cancer therapy have used a single aptamer for targeted drug delivery. However, different human cancers, such as TNBCs, particularly at late stages, are characterized by heterogeneity. Therefore, the heterogeneous nature of cancer cells is a significant issue for their success in cancer therapy. As a result, the combinational treatment that simultaneously targets several oncogenic pathways may be more effective in slowing or eliminating cancer progression. In ovarian cancer therapy, Zheng et al. [44] have investigated a bispecific aptamer's efficiency to target CD44 and EpCAM simultaneously. Their studies showed that the bispecific construct might be a promising candidate for advanced ovarian cancer. In the present study, to promote binding avidity and internalization of DOX-containing SLNs, the two aptamers (i.e., CD44 and EGFR) were simultaneously applied to form the drug delivery system. Reduction of cell viability using SLNs/DOX/Dexa/CD44/EGFR compared to SLNs/DOX/Dexa/CD44 and SLNs/DOX/Dexa/EGFR suggests that targeting numerous proliferation pathways is effective for TNBC therapy.

## 5. Conclusion

In conclusion, a surface-modified SLN, decorated with an anti-EGFR and CD-44 aptamer, was developed for targeted delivery of doxorubicin to the TNBC cell line. The obtained results indicated that dual-targeting of DOX-SLN using two aptamers is a promising approach for combination therapy. Further preclinical studies of this novel construct are underway.

## Figures and Tables

**Table 1:** Ranges and constrains.

Independent variables (factors)	Levels	
	-1	+1
Concentration ratio GMS/lecithin (A)	0.5	2.0
Amount of Tween 80 (B) (mg)	1.0	4.0
Dependent variables (responses)	Constrains	
Size (nm) (*Y*_1_)	Minimized	
PDI (*Y*_2_)	Minimized	
EE% (*Y*_3_)	Maximized	

**Table 2:** Box-Behnken experimental study (*n* = 3).

Runs	GMS/lecithin	Tween 80	Size (mean ± SD)	PDI (mean ± SD)	EE% (mean ± SD)
1	1.25	2.50	111.41 ± 34.12	0.332 ± 0.07	44.95 ± 2.04
2	1.25	4.62	69.71 ± 41.27	0.306 ± 0.03	17.67 ± 1.43
3	0.5	1.00	138.30 ± 17.96	0.361 ± 0.01	70.80 ± 1.66
4	2.31	2.50	115.01 ± 21.54	0.225 ± 0.14	86.72 ± 2.68
5	0.19	2.50	107.59 ± 31.48	0.294 ± 0.05	83.50 ± 3.39
6	2.00	4.00	80.23 ± 17.83	0.369 ± 0.08	72.38 ± 3.12
7	1.25	2.50	108.4 ± 12.49	0.336 ± 0.11	60.58 ± 1.54
8	1.25	2.50	111.82 ± 29.32	0.331 ± 0.02	50.62 ± 2.27
9	2.00	1.00	147.6 ± 19.55	0.493 ± 0.03	23.23 ± 1.94
10	1.25	0.38	214.7 ± 36.23	0.637 ± 0.01	20.23 ± 4.72
11	0.5	4.00	78.49 ± 27.31	0.191 ± 0.07	21.90 ± 2.80

**Table 3:** The characteristics of the best-fitted proposed model.

Dependent variables (responses)	Model type	*R* ^2^	Adjusted *R*^2^	Predicted *R*^2^	Adequate precision	Lack of fit (LOF)
Size (nm) (*Y*_1_)	2FI	0.7225	0.6115	0.6227	8.418	Insignificant (*p* > 0.2)
PDI (*Y*_2_)	Quadratic	0.6766	0.5599	0.5431	8.767	Insignificant (*p* > 0.1)
Log EE% (*Y*_3_)	Quadratic	0.7705	0.6453	0.6137	8.048	Insignificant (*p* > 0.4)

**Table 4:** Optimized independent variables and predicted responses.

Independent variables (factors)		Dependent variables (responses)		
GMS/lecithin	Tween 80 (mg)	Size (nm)	PDI	Log EE%
2.00	3.73	109.4	0.353	1.855 (EE% = 71.614%)

**Table 5:** Observed responses and prediction errors for model validation.

Dependent variables (responses)							
Size (nm) (*Y*_1_)		PDI (*Y*_2_)		Log EE% (log *Y*_3_)		Zeta potential (mV)	LE (%)
Observed (mean ± SD)	Error%	Observed (mean ± SD)	Error%	Observed (mean ± SD)	Error%	Observed (mean ± SD)	Observed (mean ± SD)
101.1 ± 12.6	-8.2%	0.341 ± 0.005	-3.51	1.845 ± 0.05 (EE% = 69.98 ± 7.54)	-0.54	3.9 ± 0.49	10.2 ± 1.06

**Table 6:** *In vitro* transcription protocol.

Component	Volume
DNA template	1-2 *μ*g
T7 reaction buffer	5 *μ*l
2′-F NTP mix	1.5 *μ*l
RNA polymerase	0.5 *μ*l
DEPC treated water	Up to 25 *μ*l

**Table 7:** Physicochemical properties of nanoparticles before and after aptamer decoration.

	DOX-SLN	DOX-SLN-LHON-Dexa	DOX-SLN-LHON-Dexa-EGFR/CD44
Zeta (mV) (mean ± SD)	+3.9 ± 0.49	+13.6 ± 1.83	−15.6 ± 2.07
Size (nm) (mean ± SD)	101.1 ± 12.63	147.3 ± 19.17	198.9 ± 21.34

## Data Availability

The data used to support the findings of this study are included within the article.
